# Production and Physicochemical Characterization of Cu-Doped Silicate Bioceramic Scaffolds

**DOI:** 10.3390/ma11091524

**Published:** 2018-08-24

**Authors:** Francesco Baino, Isabel Potestio, Chiara Vitale-Brovarone

**Affiliations:** 1 Department of Applied Science and Technology, Politecnico di Torino, Corso Duca degli Abruzzi 24, 10129 Torino, Italy; chiara.vitale@polito.it; 2 Lithoz CmbH, Mollardgasse 85a/2/64-69, 1060 Vienna, Austria; ipotestio@lithoz.com

**Keywords:** bioactive glass, glass–ceramic, scaffold, mesoporous, copper, angiogenesis, antibacterial, bioactivity, orbital implants

## Abstract

Development of ion-releasing implantable biomaterials is a valuable approach for advanced medical therapies. In the effort of tackling this challenge, we explored the feasibility of porous bioceramic scaffolds releasing copper ions, which are potentially able to elicit angiogenetic and antibacterial effects. First, small amounts of CuO were incorporated in the base silicate glass during melting and the obtained powders were further processed to fabricate glass–ceramic scaffolds by sponge replica method followed by sinter crystallization. As the release of copper ions from these foams in simulated body fluid (SBF) was very limited, a second processing strategy was developed. Silicate glass–ceramic scaffolds were coated with a layer of Cu-doped mesoporous glass, which exhibited favorable textural properties (ultrahigh specific surface area >200 m^2^/g, mesopore size about 5 nm) for modulating the release of copper. All the produced scaffolds, containing biocompatible crystals of wollastonite (CaSiO_3_), revealed high stability in a biological environment. Furthermore, the materials had adequate compressive strength (>10 MPa) for allowing safe manipulation during surgery. Overall, the results achieved in the present work suggest that these Cu-doped glass-derived scaffolds show promise for biomedical application and motivate further investigation of their suitability from a biological viewpoint.

## 1. Introduction

Over the last few years, understanding the biological role played by ionic dissolution products released from implantable biomaterials has become one of the most challenging topics in the context of tissue engineering applications. As many trace elements are involved in cell metabolic processes [1,2], their controlled delivery at the implant site can be exploited to elicit specific therapeutic responses in the host (patient), such as improved osteogenesis (e.g., Ca and Si [3]), angiogenesis (e.g., Co [4]) or antibacterial properties (e.g., Ag [5]).

Bioactive glasses are ideal matrices for embedding therapeutic metallic ions that can be added during the glass synthesis via either the melt-quenching route or the sol-gel method. Comprehensive overviews of these topics have been recently reported by different research groups [6,7,8,9].

Interestingly, some metallic ions can take part in multiple biological processes and, thereby, are potentially able to exert a multifunctional therapeutic action. A typical example is represented by copper, which is able to both elicit an antimicrobial effect and to stimulate angiogenesis. It has been found that copper ions are able to kill bacteria through the generation of reactive oxygen species (ROS), lipid peroxidation, protein oxidation and DNA degradation [10]. Furthermore, copper ions were shown to promote angiogenesis both in vitro and in vivo through implementing hypoxia-like conditions, which are followed by upregulation of various matrix metalloproteinases (e.g., MMP-2) and thereby result in extracellular matrix (ECM) degradation as well as the stalk endothelial cell proliferation and migration [11].

Most studies dealing with Cu-doped bioactive glasses address to applications in contact with bone; specifically, melt-derived powders [12], hierarchical macromesoporous scaffolds [13] and spray-dried nano-sized spheres [14] have been investigated. However, less popular but highly intriguing applications exist where combining improved angiogenesis with antibacterial properties is key for postoperative success, such as orbital implants in ophthalmic surgery. These ocular implants, exhibiting a spherical or egg-like shape, are inserted in the patient’s orbital socket to restore eyeball volume after surgical enucleation [15]. Whenever possible, orbital implants are sutured to the spared extraocular muscles so that the implant movement can be transmitted to an aesthetic ocular prosthesis—connected to the implant—which replicates the appearance of the healthy contralateral eye. Orbital implants should ideally remain in situ over the whole life of adult patients without undergoing any significant degradation over time; hence, clinically-used implants include non-resorbable materials such as porous hydroxyapatite, polyethylene or alumina [16]. The presence of a three-dimensional (3D) network of open macropores allows the ingrowth of fibrovascular tissue, which is key to firmly anchor the implant (scaffold) to soft orbital tissues and to provide a blood supply within the implant, thereby diminishing the risk of infection and promoting the spontaneous healing of small conjunctival exposures [17]. Despite all these advantages, commercial porous orbital implants still suffer from a non-negligible failure rate [18] and are highly expensive, thereby often pushing patients to choose other cheaper solutions, such as solid polymeric spheres that, however, do not allow fibrovascular ingrowth and are potentially susceptible to a higher risk of infection. 

Therefore, there is the need for developing novel porous orbital implants that could lead to better performances compared to the existing options. This challenge is tackled in the present work that reports the feasibility and physicochemical characterization of Cu-doped porous glass–ceramic scaffolds and discusses their potential suitability as orbital implant materials. It is worth underlining that, for this purpose, we tried to fulfil two apparently irreconcilable requirements, i.e., the need for a permanent material combined with the release of copper ions over time.

## 2. Materials and Methods

The basic material used for scaffold preparation was a melt-derived silicate glass belonging to the 57SiO_2_-34CaO-6Na_2_O-3Al_2_O_3_ (mol. %) system (SCNA). This choice was performed based on the primary requirement needed for an orbital implant, i.e., the absence of significant degradation over time during contact with biological fluids. SCNA, initially developed for osseous applications and exhibiting an almost-inert behavior in vitro [19], was thought potentially suitable for use as orbital implant material, too.

Copper was embedded in the SCNA-derived scaffolds through two different approaches: (i) incorporation of copper during the melting of the glass that will be used to produce scaffolds; or (ii) coating of the scaffold with a Cu-containing sol-gel derived mesoporous bioactive glass (xCu-MBG, x = 1 or 5 mol. %) layer.

### 2.1. First Strategy: Preparation of Scaffolds Based on Cu-Containing Melt-Derived Glasses Ubsection

The SCNA-based glass-forming batches were prepared by mixing analytical grade SiO_2_, Al_2_O_3_, CaCO_3_ and Na_2_CO_3_ along with proper amounts of CuO (all purchased from Sigma-Aldrich, St. Louis, MO, USA). Copper-doped glasses were obtained by varying the CaO-to-CuO molar ratio. Specifically, two Cu-doped glasses were produced, i.e., SCNA-2Cu (57SiO_2_-32CaO-6Na_2_O-3Al_2_O_3_-2CuO mol. %) and SCNA-5Cu (57SiO_2_-29CaO-6Na_2_O-3Al_2_O_3_-5CuO mol. %). The raw materials (powders) were weighed and subsequently mixed in an orbital shaker to ensure adequate homogenization of the blends. The mixtures of oxides and carbonates were placed in alumina crucibles with a lid and heated up to 1550 °C for about 30 min in an electric furnace; then, the crucibles were taken out of the furnace and the melt was quenched in cold water to obtain glass frits.

After being dried at room temperature, the glass was placed in a zirconium oxide milling jar and pulverized in a planetary mill (Pulverisette 6, Fritsch, Idar-Oberstein, Germany) at 300 rpm for a total time of 70 min. Afterwards, the powders were manually sieved through stainless steel sieves (Giuliani Technologies, Torino, Italy) in order to obtain a final particle size below 32 μm.

Porous SCNA, SCNA-2Cu and SCNA-5Cu scaffolds were fabricated from respective starting glasses via sponge replica method. Briefly, this technique involves the impregnation of an open-cell polymeric sponge with a slurry containing glass particles and a binder in order to coat the walls and struts of the sponge with a thin layer of glass. The sponge is then dried and the resulting green body is heat-treated in order to eliminate the polymeric template and to sinter and (partially) crystallize the glass particles.

Specifically, the glass suspensions were prepared by mixing glass powders (40 wt. %), polyvinyl alcohol (PVA) (6 wt. %) and distilled water (54 wt. %) along with a small amount (0.1 wt. % of total solid loading) of a dispersant/deflocculant agent (Dolapix CE 64, Zschimmer and Schwarz GmbH, Lahnstein, Germany). First, PVA was dissolved in water under magnetic stirring (200 rpm) for 1 h at 90 °C; at the end of this stage, the same amount of liquid water as that evaporated during mixing was re-added to the batch in order to maintain the initial mass ratio, then, glass powders and Dolapix were dispersed into the batch that was stirred for 0.5 h to obtain a homogeneous slurry.

A commercial open-cell polyurethane (PU) sponge of 45 ppi (pores per inch) was manually cut into 10 mm × 10 mm × 10 mm cuboids that were dipped into the slurry. After withdrawal, the excess slurry was removed by cycles of compression (20 kPa for 1 s) to reduce the sponge thickness up to 60% along the three spatial directions. This infiltration-compression cycle was repeated three times; then, the glass-coated polymeric cuboids were left to dry overnight at room temperature and heated up to 1000 °C (SCNA-2Cu) or 950 °C (SCNA-5Cu) for 3 h in an electric furnace in air (heating rate 5 °C/min) to burn out the PU foam and sinter the glass particles.

### 2.2. Second Strategy: Scaffolds Coated with xCu-MBGs

The process used for the synthesis of xCu-MBG was a sol-gel-type route commonly referred to as evaporation-induced self-assembly (EISA) method. xCu-MBG were prepared by incorporating CuO to partially replace CaO in the MBG composition (molar ratio Si/Ca/Cu = 80/19/1 and 80/15/5 for 1Cu-MBG and 5Cu-MBG, respectively).

The classical synthesis procedure reported by Yan et al. [20] was followed as a base reference and properly modified to incorporate copper in the mesoporous glass. The synthesis involved the use of a commercially available surfactant (Pluronic P123) as a structure-directing agent. Tetraethyl orthosilicate (TEOS) and calcium nitrate tetrahydrate Ca(NO_3_)_2_·4H_2_O were used to supply SiO_2_ and CaO, respectively, while CuO was sourced as CuCl_2_ (all the reagents were purchased from Sigma-Aldrich, St. Louis, MO, USA). Briefly, 4.0 g of P123 were dissolved in 60.0 g of ethanol with 1.0 g of 0.5 M HCl under constant stirring at room temperature. Once P123 was completely dissolved, the glass precursors were slowly added in the following order: 6.7 g of TEOS, 1.8 or 1.425 g of Ca(NO_3_)_2_·4H_2_O (for 1Cu-MBG or 5Cu-MBG, respectively), and 0.054 or 0.27 g of CuCl_2_ (for 1Cu-MBG or 5Cu-MBG, respectively).

The xCu-MBG coatings were applied on SCNA scaffolds by completely immersing them into the sol for 10 min. After withdrawal, the scaffolds were left to dry at room temperature for 24 h. This immersion-drying cycle was repeated for three time. Eventually, the scaffolds were calcined at 650 °C for 5 h in air (heating and cooling rate of 2 °C/min and 5 °C/min, respectively) to obtain xCu-MBG-coated SCNA scaffolds.

### 2.3. Characterizations

#### 2.3.1. Hot-Stage Microscopy

The hot-stage microscopy (HSM) measurements were carried out on Cu-doped melt-derived glasses by using a heating stage optical microscope equipped with image analysis system (Expert System Solutions, Modena, Italy). SCNA-2Cu and SCNA-5Cu cylindrical specimens suitable for the analysis (diameter 2 mm, height 3 mm) were obtained by manually pressing the glass powders (particle size < 32 μm) in a stainless steel mould and then positioned onto a high-purity alumina plate. Black-and-white images (silhouettes) of the profile of the specimens were taken from 25 to 1400 °C with a heating rate of 10 °C/min. The variation of the sample dimensions upon heating were measured and the shrinkage (%) was quantified, along with the temperature of first shrinkage (T_FS_) and maximum shrinkage (T_MS_). This analysis was key to select the appropriate sintering temperature for fabricating the scaffolds.

#### 2.3.2. Microstructural Analysis

Powdered materials underwent X-ray diffraction (XRD) by using a X’Pert Pro PW3040/60 diffractometer (PANalytical, Eindhoven, The Netherlands) operating at 40 kV and 30 mA with Bragg-Brentano camera geometry and Cu Kα incident radiation (wavelength λ = 0.15405 nm). Identification of crystalline phases was performed by using X’Pert HighScore software (2.2b) equipped with the PCPDFWIN database (http://pcpdfwin.updatestar.com).

Wide-angle XRD (2θ within 10–70°) was performed on melt-derived materials (SCNA-2Cu and SCNA-5Cu), while small-angle XRD (2θ within 0.9–5°) was applied to assess the presence of an ordered mesoporous structure in the mesoporous coating.

#### 2.3.3. Morphology, Composition and Porosity

Scanning electron microscopy (SEM; Supra^TM^ 40, Zeiss, Oberkochen, Germany) was used to evaluate the morphology of both scaffolds and coatings as well as the pore/strut characteristics of the scaffolds. Compositional analysis was performed by energy dispersive spectroscopy (EDS), which was included in the SEM equipment. The samples were silver-coated prior to the analysis and inspected at an accelerating voltage in the range of 5 to 15 kV.

The 3D pore/strut architecture of the scaffolds was also investigated by means of X-ray micro-computed tomography (micro-CT). Scans were performed by using a commercial micro-CT unit Skyscan 1174.v2 (SkyScan-Bruker, Kontich, Belgium). The major scanning parameters included fixed rotation step 0.3°, image pixel size 7.6 μm, exposure time 8500 ms, and 500-μm thick aluminum filter. 3D reconstruction and visualization were performed by using the NRecon and DataViewer/CTVox softwares (SkyScan-Bruker, Kontich, Belgium), whereas the CTAn program (Bruker, Billerica, MA, USA) was employed for 2D/3D processing and quantitative analysis of data.

Textural parameters of mesoporous coatings were assessed by nitrogen (N_2_) adsorption-desorption porosimetry measurements performed at −196 °C (Quantachrome Autosorb1, Quantachrome, Boynton Beach, FL, USA). The specific surface area (SSA) was assessed by using the Brunauer-Emmet-Teller (BET) method [21], and the pore size distribution (along with the mean pore size) was determined through the density functional theory (DFT) isotherm reconstruction approach [22]. The mesoporous structure was also studied by means of scanning-transmission electron microscopy (STEM).

#### 2.3.4. Mechanical Testing

The compressive strength of scaffolds was evaluated by means of crushing tests (Syntech 10/D machine, MTS, Eden Prairie, MN, USA; cross-head speed set at 0.5 mm min^−1^) on polished cuboids as L_C_/A_C_, where L_C_ (N) is the maximum compressive load registered during the test and A_C_ (mm^2^) is the resistant cross-sectional area perpendicular to the load axis. Results are expressed as mean ± standard deviation calculated on at least five samples for each type.

#### 2.3.5. Copper Release Studies

Release of copper ions from the scaffolds soaked in simulated body fluid (SBF) was assessed by means of inductively-coupled plasma-mass spectrometer (ICP-MS; iCAP™ Q spectrometer, ThermoFisher Scientific, Waltham, MA, USA). SBF was prepared according to the protocol recommended by Kokubo and Takadama [23]. The scaffolds (triplicate samples) were maintained in polyethylene bottles filled with SBF at 37 °C in an incubator under static conditions; the copper release was monitored up to 30 days. A mass-to-volume ratio of 1.5 mg/mL was used, as recommended in previous studies [24]. At each time point, 1 mL aliquot of solution was collected and replaced with fresh SBF, as suggested in [24]. At the end of the experiment, the samples were extracted, rinsed with ultra-pure water, left to dry overnight at room temperatures and analyzed by SEM-EDS. The solution aliquots collected at each time point were analyzed by ICP-MS to determine copper elemental concentration.

## 3. Results and Discussion

### 3.1. Scaffolds Based on Cu-Containing Melt-Derived Glasses

**Figure 1:**
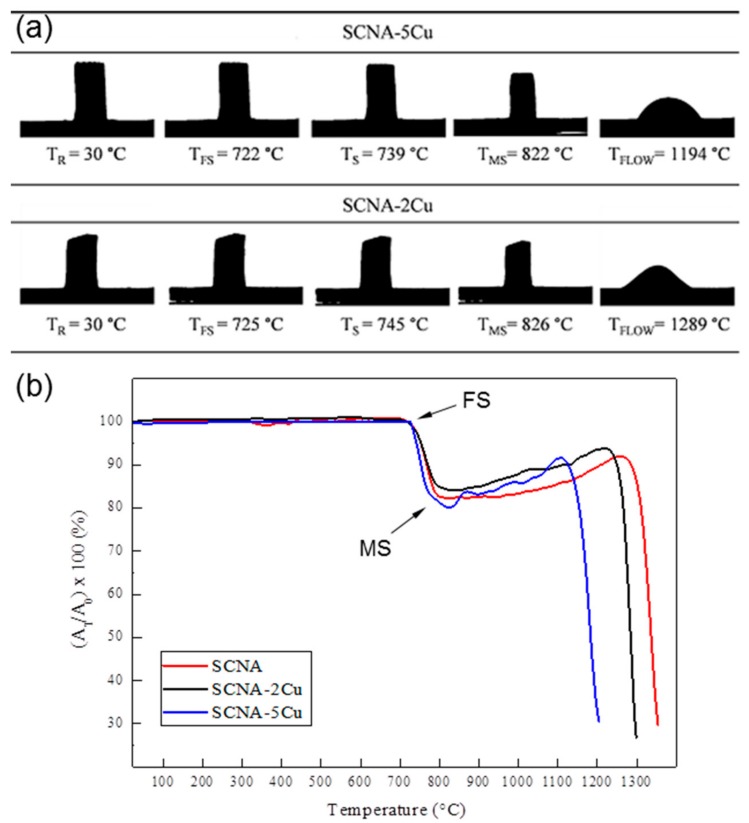
Results of HSM: (**a**) silhouettes of the Cu-doped SCNA-based glasses sample at different characteristic temperatures upon heating (T_R_ room temperature, T_FS_ first shrinkage, T_S_ softening, T_MS_ maximum shrinkage, T_FLOW_ flowing); (**b**) shrinkage variation as a function of temperature (A_0_ area of the silhouette at room temperature, A_T_ sample area at the current temperature).

HSM analysis guided the selection of the sintering treatment for the fabrication of SCNA-2Cu and SCNA-5Cu scaffolds. As shown in Figure 1a, SCNA-2Cu and SCNA-5Cu samples maintained their unaltered cylindrical shape up to 722 and 725 °C, respectively, when first shrinkage occurred. Maximum shrinkage was reached within 820–830 °C and the samples underwent melting above 1190 °C. Figure 1b compares the thermal shrinkage of melt-derived Cu-doped glasses with that of the parent SCNA material. The thermal behavior of the three glasses is similar as they all exhibit a one-stage shrinkage upon heating, and the maximum shrinkage is followed by a plateau before reaching the melting temperature. The first shrinkage temperature does not significantly change with CuO content. Interestingly, decrease of the melting temperature was observed with the increasing amount of CuO in substitution to CaO in the glass composition. This result is in accordance with those obtained by Wers et al. [25]. CuO is known to be an intermediate oxide but, if there is a relatively low amount of alkaline ions in the glass, Cu^2+^ participates only as a network modifier assuming the same role as Ca^2+^ [26]. Therefore, the reduction of melting temperature with increasing amount of CuO can be related to the difference of high-temperature stability of CaO and CuO. CaO is a widely accepted refractory compound, conversely to CuO (the refractoriness of a compound reflects its melting point, and in the case of CaO and CuO melting points are 2590 and 1326 °C, respectively). Based on the HSM results, the selected sintering temperatures for making the scaffolds were 1000 and 950 °C for SCNA-2Cu and SCNA-5Cu, respectively (well above the maximum shrinkage and in the middle of the densification region).

**Figure 2:**
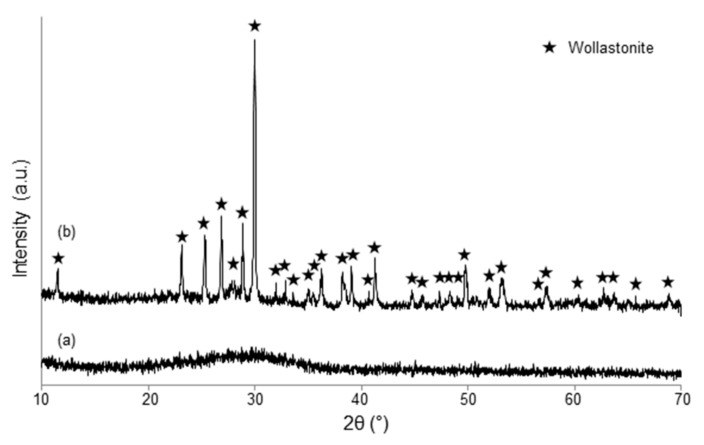
Wide-angle XRD pattern of (**a**) as-quenched SCNA-2Cu and (**b**) SCNA-2Cu thermally treated at 1000 °C for 3 h.

**Figure 3:**
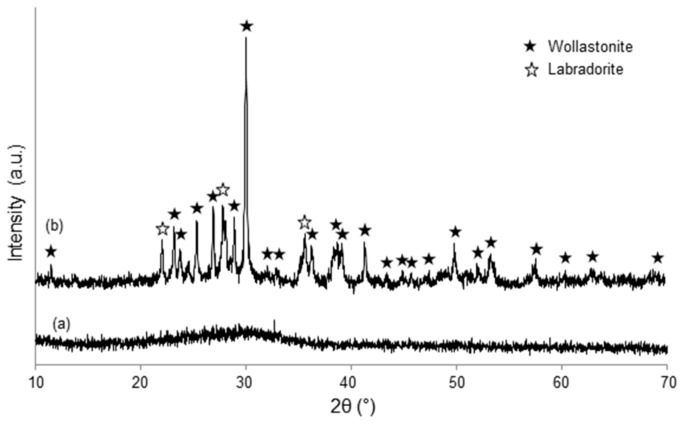
Wide-angle XRD pattern of (**a**) as-quenched SCNA-5Cu and (**b**) SCNA-5Cu thermally treated at 950 °C for 3 h.

As-poured SCNA-2Cu and SCNA-5Cu are completely amorphous materials (glasses), as demonstrated by the broad halo (2θ within 20–40°) in the wide-angle XRD patterns shown in Figure 2a and Figure 3a. Analogous results were found elsewhere for the parent SCNA material [27]. Therefore, it can be stated that the introduction of CuO in the glass matrix does not affect the amorphous character of the glasses. The wide-angle XRD analyses performed on SCNA-2Cu and SCNA-5Cu scaffolds ground in powders (Figure 2b and Figure 3b) indicate that wollastonite (CaSiO_3_; PDF code: 00-027-0088) is the major crystalline phase developed in both materials upon heat treatment. This is consistent with previous observations on Cu-free crystallized SCNA [27] as well as with the results reported by Pérez et al. (devitrification of a SiO_2_-CaO-Na_2_O-Al_2_O_3_ glass to wollastonite within 850–950 °C) [28]. The biocompatibility of wollastonite is well known and recognized since the 1980s [29,30]. Labradorite (Ca_0.65_Na_0.35_(Al_1.65_Si_2.35_O_8_); PDF code: 01-083-1370) was also detected as a secondary crystalline phase in heat-treated SCNA-5Cu. In summary, both scaffolds are actually made of glass–ceramic materials.

**Figure 4:**
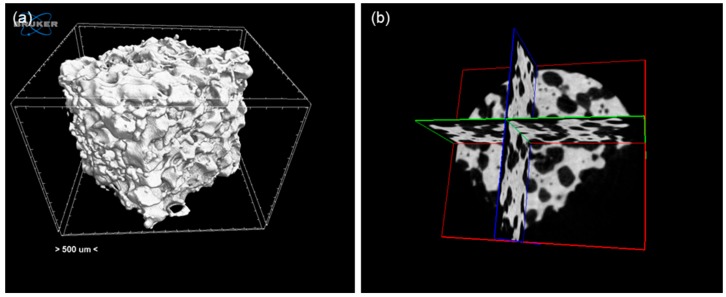
Exemplary micro-CT analysis of the Cu-doped scaffolds: (**a**) 3D reconstruction of the scaffold volume and (**b**) cross-sections in the [xy], [xz] and [yz] orthogonal planes.

Micro-CT investigations allowed visualizing the pore/strut structure of the scaffolds (Figure 4), characterized by a 3D network of open and interconnected macropores with a size in the range of 450–500 µm (total porosity about 50 vol. %). The connectivity density of the scaffolds is around 3.0 mm^−3^ which, interestingly, is very close to that of femur trabecular bone from human patients [31,32]. In general, pore interconnectivity is a key feature for implantable scaffolds to allow biological fluid perfusion, cell colonization and blood vessel access. A comparison with the pore characteristics of commercial implants supports the architectural suitability of Cu-doped SCNA-based scaffolds for orbital applications (for example, the total porosity and pore size of Medpor^®^ polyethylene implant are within 30–70 vol. % and 100–1000 µm, while the bioceramic alumina implant has a nominal pore size centered at 500 µm [33]).

Compressive strength of SCNA-2Cu and SCNA-5Cu scaffolds (23 ± 2 and 21 ± 3 MPa, respectively) is enough to allow safe manipulation of the implants during surgical procedures.

**Figure 5:**
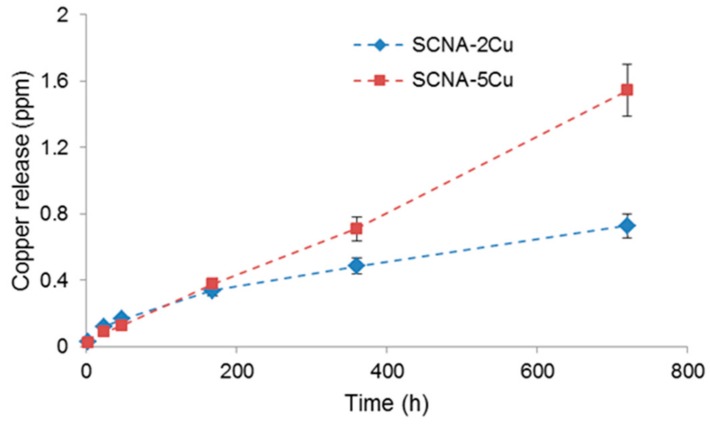
Profiles of copper release from SCNA-2Cu and SCNA-5Cu scaffolds following immersion in SBF.

Copper ion release from SCNA-2Cu and SCNA-5Cu scaffolds as a function of immersion time in SBF is shown in Figure 5. In general, a continuous release over time was observed and the amount of Cu ions in SBF increased with increasing amount of Cu in the as-prepared glass (SCNA-5Cu > SCNA-2Cu). A quantitative evaluation of in vitro release of copper is essential in order to rationalize the effective therapeutic properties of the materials. The therapeutic behavior, in fact, will depend on the amount of copper ions released. Most of the studies existing in the literature reveal that an antimicrobial effect can be obtained with a copper ion concentration within 50–100 ppm [13,34,35], which is significantly higher compared to the values achieved for SCNA-2Cu and SCNA-5Cu. A similar situation was observed as far as the angiogenetic effect is concerned: a clear promotion of angiogenesis was reported in vitro and in vivo when the level of copper released is on the order of a few tens of ppm [13,36].

Detection of no mass loss at the end of the dissolution experiments for both types of scaffolds further confirms the high stability of SCNA-2Cu and SCNA-5Cu in a biological environment, which can be interesting when non-resorbable implants are needed (e.g., permanent orbital implants).

The high stability of these scaffolds in SBF and the low release of copper are dictated by the basic SCNA composition. In principle, other less-stable melt-derived glasses could be selected and doped with copper for improving the release of copper to therapeutic levels, thus eliciting acceptable antibacterial and pro-angiogenic actions. However, these glass-derived materials would undergo progressive dissolution over time, which contradicts the main requirement of orbital implants (high stability and structural integrity). Therefore, a completely different approach was investigated based on the deposition of reactive but thin glass coatings on nearly-inert glass–ceramic scaffolds. 

### 3.2. Scaffolds Coated with Cu-Doped MBGs

In order to increase the release of copper from the scaffolds, a second approach was pursued involving the coating of SCNA scaffolds with a Cu-doped MBG layer. In fact, the mesoporous texture and high SSA of mesoporous materials are known to intensify the rate of surface reactions compared to melt-derived ones, thus leading to a faster release of therapeutic agents during glass dissolution [37].

**Figure 6:**
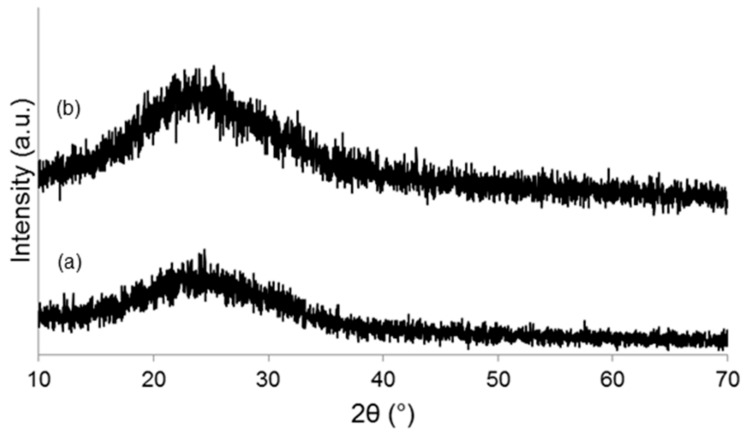
Wide-angle XRD pattern of (**a**) 1Cu-MBG and (**b**) 5Cu-MBG.

**Figure 7:**
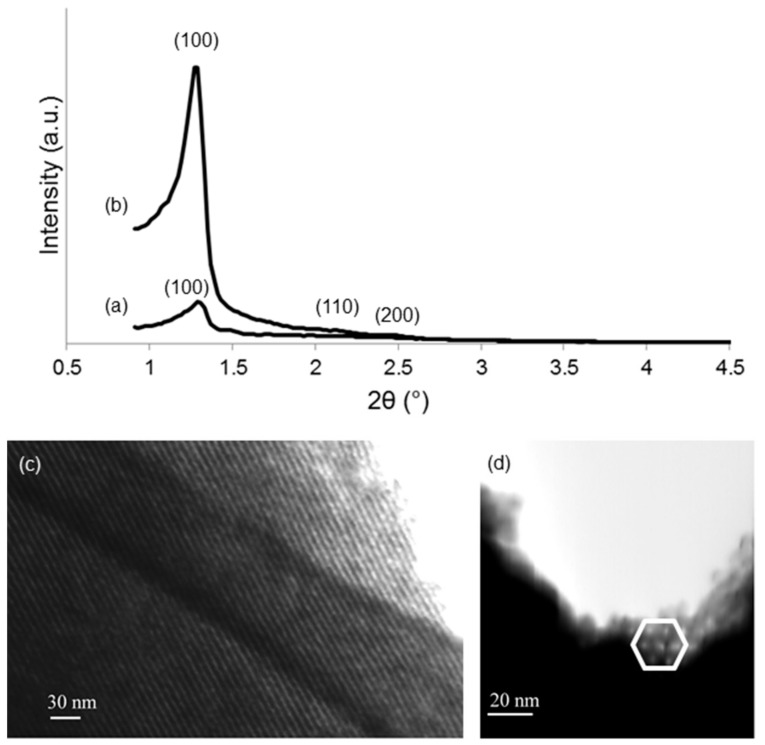
Mesoporous structures of Cu-doped MBGs: (top) small-angle XRD pattern of (**a**) 1Cu-MBG and (**b**) 5Cu-MBG; (bottom) STEM images of 5Cu-MBG recorded along (**c**) [100] and (**d**) [001] directions. In (**d**) the typical hexagonal symmetry of the mesopores is emphasized.

The wide-angle XRD diffraction patterns of calcined 1Cu-MBG and 5Cu-MBG are shown in Figure 6 and reveal the completely amorphous nature of both glasses, as demonstrated by the sole presence of a broad halo (2θ within 15–35°). On the contrary, in the small-angle regime (Figure 7a,b), both glasses show the diffraction peaks typical of the scattering patterns of a two-dimensional hexagonal p6mm lattice [20]. The d_100_ is about 6.8 nm for both materials and, assuming a 2D hexagonal symmetry, the cell parameter results to be 7.8 nm. The low intensities for the reflections of 1Cu-MBG is probably due to the lower order of the mesostructure compared to 5Cu-MBG. According to the available literature, the effects of copper ions on the textural properties of MBGs still are under debate. Although it was well shown that doping with copper leads to a decrease of SSA compared to mesoporous pure silica, clear relationships between the increasing content of copper and the mesostructural order, SSA and mesopore size remain to be fully understood [13,14].

STEM images of 5Cu-MBG along the [100] and [001] directions allow the visualization of a highly-ordered hexagonal arrangement of 1D parallel channels (Figure 7c,d), in agreement with small-angle XRD results.

**Figure 8:**
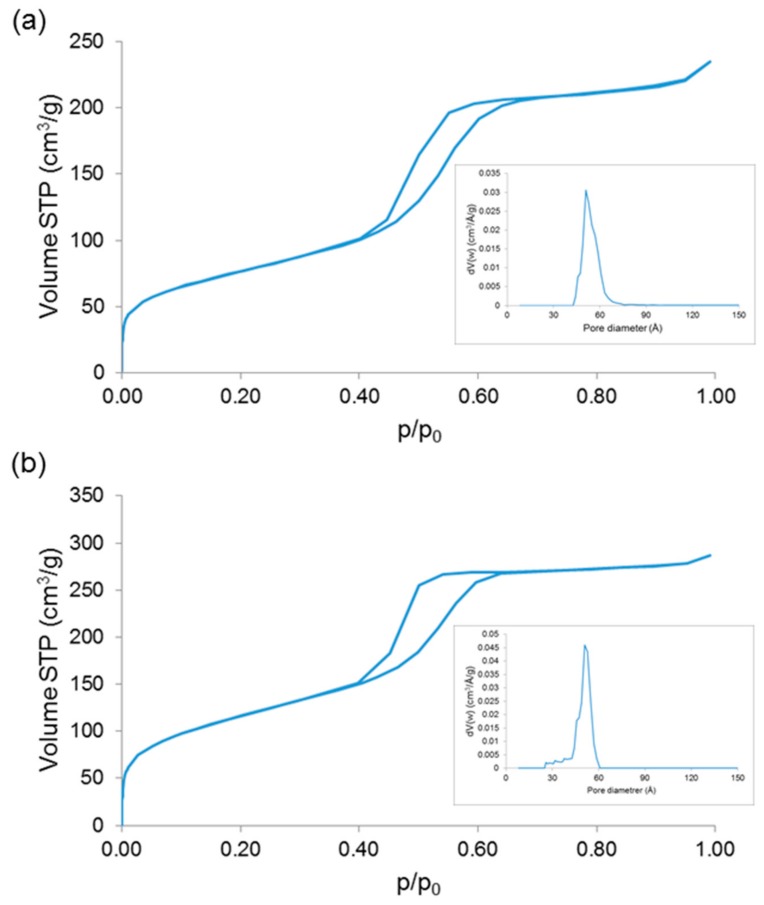
Nitrogen adsorption-desorption isotherms of (**a**) 1Cu-MBG and (**b**) 5Cu-MBG. Insets in both pictures report the pore size distribution.

N_2_ adsorption-desorption measurements further confirmed the mesoporous texture of 1Cu-MBG and 5Cu-MBG as both materials exhibited a type-IV isotherm pattern (Figure 8), which is associated to nanopores within 2–50 nm [38]. The shape of the hysteresis loop, which can provide information about the shape of the mesopores [39,40], reveals the presence of uniform mesopores of approximately cylindrical shape with MCM41-like hexagonal symmetry (H2-type loop). The textural characteristics are collected in Table 1; these results are consistent with those assessed for the parent SiO_2_-CaO-P_2_O_5_ ternary MBGs [20,41] and suggest that the SSA can be increased by increasing the CuO content in the glass, which is in agreement with the trend observed by Wu et al. about Cu-doped MBGs [13].

Pore characteristics assessed by micro-CT were in line with previous investigations on SCNA scaffolds [42]. Interestingly, the presence of the MBG coating induced no significant variations in terms of macropore size (about 500 µm before and after the coating procedure) while the total porosity moderately increased after the surface treatment (60 vs. 50 vol. %). This difference can be explained considering that, in order to apply the MBG coating, SCNA scaffolds were immersed in a strongly acidic sol having very low value of pH (1.04 and 0.85 for 1Cu-MBG and 5Cu-MBG sols, respectively). The strong acidity of the sol may be responsible for the surface erosion of scaffold struts, which resulted in a higher porosity. This had an impact on the mechanical properties, too: in fact, the compressive strength of both Cu-MBG-SCNA scaffolds was 10.0 ± 2.0 MPa, which is about half as lower as the compressive strength obtained for uncoated SCNA samples (23.0 ± 2.0 MPa) manufactured through the same procedure. If application as orbital implant material is a goal, these values of mechanical strength are enough to allow safe manipulation during surgery as well as postoperative integrity. Comparison with commercial implants is not possible due to the lack of available data in the literature. Problems of mechanical integrity were mentioned—albeit without providing quantitative data—for bovine hydroxyapatite orbital implants [43] that, however, are significantly more porous (80 vol. %) than the materials developed in the present work.

**Figure 9:**
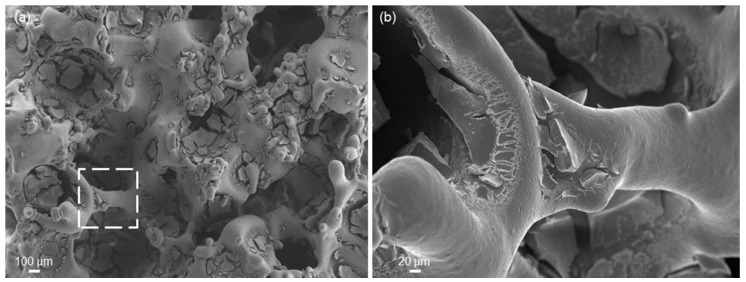
SEM micrographs of (**a**) the porous structure of 5Cu-MBG-SCNA scaffold (magnification 100×) and (**b**) detail of a trabecula (dashed square in (**a**)) coated by the MBG layer (magnification 500×).

Figure 9 shows SEM images at different magnifications of 5Cu-MBG-SCNA. In Figure 9a, the 5Cu-MBG layer is shown to coat quite uniformly the pore walls of the scaffold. As displayed in Figure 9b, the MBG coating appears to cover all the surface of the single struts lying underneath; however, some defects and cracks can be observed in the coating, which suggest the need for further optimization of the deposition procedure and/or calcination treatment to produce flawless glass layers.

The pore/strut architecture of a given scaffold clearly dictates the potential suitability for a specific application. The total porosity and macropore size of MBG-coated materials are adequate for use in the field of anopthalmic socket surgery, being comparable to those of commercially-available and clinically-used orbital implants (see the considerations already presented for SCNA-2Cu and SCNA-5Cu scaffolds in the Section 3.1).

**Figure 10:**
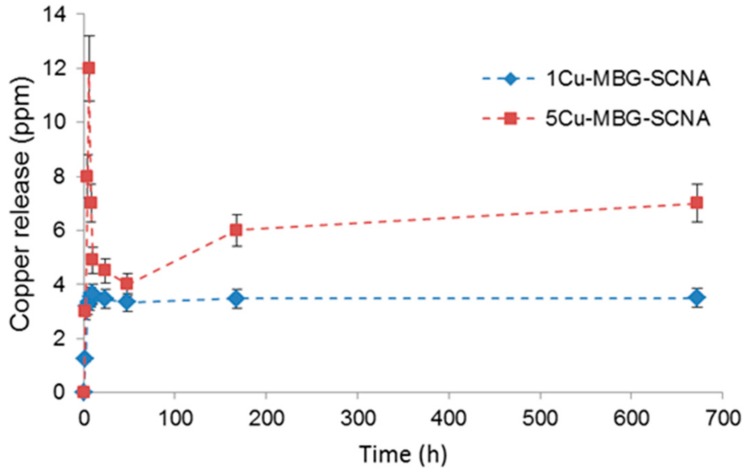
Profiles of copper release from 1Cu-MBG-SCNA and 5Cu-MBG-SCNA scaffolds following immersion in SBF.

The release of copper ions from 1Cu-MBG-SCNA and 5Cu-MBG-SCNA scaffolds as a function of immersion time in SBF is shown in Figure 10. The trend of copper ion release can be split into three parts: (i) initial increase in the first hours of immersion; (ii) drop observed around 48 h; and (iii) a plateau stage (1Cu-MBG-SCNA) or a slight increase till 2 weeks (5Cu-MBG-SCNA). The drop of copper levels might be due to the precipitations of copper in the hydroxyapatite layer that is formed on the surface of both scaffolds upon immersion in SBF. In fact, it is known that hydroxyapatite can act as a cation exchanger and may incorporate metallic ions such as Zn, Co and Cu in its structure [44].

Interestingly, weight measurements of scaffolds before and after in vitro tests (2 weeks) revealed no mass variation during immersion in SBF. Furthermore, pH measurements performed on the testing solution just revealed a slight variation towards alkalinity (from 7.4 to 7.6).

From these results it is clear that the samples are able to deliver copper ions and that this release depends on the initial concentration of copper in the MBG formulation. Altogether, the release of copper ions from 5Cu-MBG-SCNA scaffolds is one order of magnitude larger than that from melt-derived SCNA-2Cu and SCNA-5Cu at each time point (Figure 10 vs. Figure 5). On the contrary, when results from 1Cu-MBG-SCNA are compared with those from melt-derived SCNA-2Cu and SCNA-5Cu, copper ion release appears larger at each time point, yet the values are of the same order of magnitude. This further confirms that the high SSA of the mesoporous coating facilitates the delivery capacity of the therapeutic metallic agent. The concentrations of copper released from 5Cu-MBG-SCNA could be potentially effective to elicit antiseptic and angiogenetic effects [13,34,35], thereby encouraging further biological investigation on this material.

**Figure 11:**
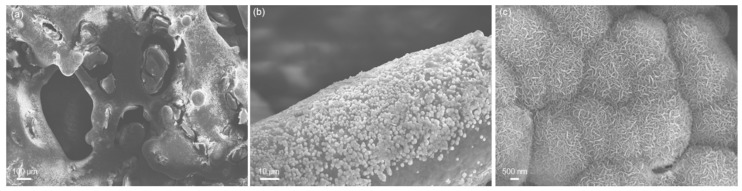
SEM micrographs of 5Cu-MBG-SCNA scaffold after 15 days of immersion in SBF: (**a**) overview of the macroporous structure (magnification 200×); (**b**) newly formed globular agglomerates on the surface of a trabecula (magnification 3000×); and (**c**) nanocrystalline nature of this hydroxyapatite-like phase (magnification 30,000×) characterized by needle-shaped crystals organized according to a typical cauliflower morphology.

Figure 11 shows exemplary SEM images of a 5Cu-MBG-SCNA scaffold after 15 days of immersion in SBF at different magnifications. The pore walls and struts of the scaffolds are coated by a newly formed phase characterized by a cauliflower morphology, i.e., globular agglomerates formed by needle-like nanocrystals. Compositional analysis reveals that this phase is a calcium phosphate with a Ca-to-P atomic ratio of 1.68, which is very close to that of stoichiometric hydroxyapatite (1.67). It is worth underlining that, during hydroxyapatite formation, just the scaffold surface (i.e., the thin surface layer of MBG) reacts with the biological fluids, while the SCNA skeleton exhibits nearly inert behavior [45]; this fulfils the primary requirement of orbital implant materials, i.e., the stability over time to allow orbital volume filling and adequate support to surrounding orbital structures.

If apatite-forming ability is unnecessary for orbital implant materials, on the other hand the bioactivity of the Cu-doped MBG-SCNA scaffold would be key for applications in other biomedical fields, such as bone tissue repair. Therefore, Cu-doped MBG-SCNA scaffolds show promise for use in contact with hard tissues to regenerate bone, ensuring structural support at the defect site (due to the strong SCNA skeleton) as well as promoting bone in-growth and safe anchorage to surrounding host tissues (due to the MBG layer).

## 4. Conclusions

Copper, which is known as a proangiogenic and antimicrobial agent, was incorporated into glass-derived scaffolds via two different approaches. The intended application was in the field of permanent orbital implant materials and, accordingly, the base material for making scaffolds was an almost-inert silicate glass. Melt-derived Cu-doped strong macroporous scaffolds were successfully produced by sponge replication, but the release of copper was inadequate to elicit a therapeutic effect. The second strategy, involving the deposition of a Cu-doped mesoporous glass layer on the scaffold struts, allowed achieving a more sustained release of copper, which motivates further research on this topic. Furthermore, the bioactive glass-coated scaffolds exhibited a good apatite-forming ability upon immersion in biological fluids, which could suggest promising and interesting applications in other biomedical areas like bone tissue engineering.

## Figures and Tables

**Table 1:** Textural parameters obtained by nitrogen adsorption-desorption porosimetry for the calcined mesoporous materials.

Sample	SSA (m^2^/g)	Mean Pore Size (nm)
1Cu-MBG	275	5.1
5Cu-MBG	432	5.1
